# Zinc Affects Differently Growth, Photosynthesis, Antioxidant Enzyme Activities and Phytochelatin Synthase Expression of Four Marine Diatoms

**DOI:** 10.1100/2012/982957

**Published:** 2012-05-03

**Authors:** Thi Le Nhung Nguyen-Deroche, Aurore Caruso, Thi Trung Le, Trang Viet Bui, Benoît Schoefs, Gérard Tremblin, Annick Morant-Manceau

**Affiliations:** ^1^Mer, Molécules, Santé, EA 2160, LUNAM Université, Faculté des Sciences et Techniques, Université du Maine, Avenue Olivier Messiaen, 72085 Le Mans cedex 9, France; ^2^Laboratory of Plant Physiology, Department of Biology, University of Education of Ho Chi Minh City, 5th District, 280 An Duong Vuong, Ho Chi Minh City, Vietnam; ^3^Plant Physiology Department, Faculty of Biology, University of Natural Sciences, 227 Nguyen Van Cu, 5th District, Ho Chi Minh City, Vietnam

## Abstract

Zinc-supplementation (20 **μ**M) effects on growth, photosynthesis, antioxidant enzyme activities (superoxide dismutase, ascorbate peroxidase, catalase), and the expression of phytochelatin synthase gene were investigated in four marine diatoms (*Amphora acutiuscula*, *Nitzschia palea*, *Amphora coffeaeformis* and *Entomoneis paludosa*). Zn-supplementation reduced the maximum cell density. A linear relationship was found between the evolution of gross photosynthesis and total chlorophyll content. The Zn treatment decreased the electron transport rate except in *A. coffeaeformis* and in *E. paludosa* at high irradiance. A linear relationship was found between the efficiency of light to evolve oxygen and the size of the light-harvesting antenna. The external carbonic anhydrase activity was stimulated in Zn-supplemented *E. paludosa* but was not correlated with an increase of photosynthesis. The total activity of the antioxidant enzymes did not display any clear increase except in ascorbate peroxidase activity in *N. palea*. The phytochelatin synthase gene was identified in the four diatoms, but its expression was only revealed in *N. palea*, without a clear difference between control and Zn-supplemented cells. Among the four species, *A. paludosa* was the most sensitive and *A. coffeaeformis*, the most tolerant. *A. acutiuscula* seemed to be under metal starvation, whereas, to survive, only *N. palea* developed several stress responses.

## 1. Introduction

Marine diatoms fulfill important roles in the biosphere. Among these, diatoms are responsible for about 25% of annual inorganic carbon fixation in oceans [1]. This CO_2_ is fixed through the photosynthetic process into energy-rich molecules that ultimately serve to feed the other levels of the trophic networks. To fulfill this role, diatoms as other living organisms must find in their environment good conditions, including the right range of macro- and microelements. Among the mandatory microelements required for cell functioning, zinc (Zn) occupies a particular place because it acts as a structural component [2] and as functional component of numerous enzymes, in some gene transcription regulators [3] and as a cofactor in zinc-finger protein involved in mitosis regulation [4] (for review, see [5]). As for other nutrient, Zn should be present within a definite range to allow optimum cell functioning and growth. In Zn-deficient conditions, diatoms cannot develop whereas when Zn is present in excess, crucial processes are inhibited partially or totally (growth: [6–8], photosynthesis: [9, 10]) while the oxidative stress develops [11–13]. Because the optimal range of Zn concentrations depends on diatom species, this type of algae is used as bioindicators [14].

Physiological and biochemical studies have demonstrated that the capacity to tolerate Zn is linked to the ability to establish defense mechanisms (for reviews see [5, 15]). Among these mechanisms, Zn chelation seems to be major. Zn ions can be chelated by exopolysaccharides as in the diatom *Skeletonema costatum* [16] or in the cytoplasm by phytochelatins, which are cysteine-rich pseudopeptides. Phytochelatins are synthesized by addition of glutathione units (*γ*-Glu-Cys-Gly) through the catalytic action of phytochelatin synthase (PCS), a *γ*-glutamyl cysteine transpeptidase [17].

The aim of this study was to compare the effect of an increase of Zn ion concentration on the growth, photosynthetic process, and responses to metal stress of four diatom species. *Amphora acutiuscula* and *Nitzschia palea* were harvested and isolated from the South-East Vietnamese coast, at the Can Gio site, which is confronted by pollution from the Mekong River, and two other diatom species (*A. coffeaeformis* and *Entomoneis paludosa*) isolated from the French Atlantic coast. *N. palea* often develops in polluted waters [18], and *A. coffeaeformis* has been shown to be a tolerant species to UV [19, 20] and Cu [10] but sensitive to Cd [14].

## 2. Materials and Methods

### 2.1. Culture Conditions

*Amphora acutiuscula* Kützing and *Nitzschia palea* (Kützing) Smith were collected at the Can Gio coastal site in South East Vietnam (latitude: 10°40′09′′; longitude: 107°00′59′′), whereas *A. coffeaeformis* (Agardh) Kützing and *Entomoneis paludosa* (W. Smith) Reimer were collected on the French Atlantic coast and were obtained from the Nantes Culture Collection (strains UTC58 and NCC18.2, resp.). Each taxon was axenically cultured in artificial seawater (ASW) prepared from Millipore ultrapure water according to Harrison et al. [21]. Diatoms originating from the Vietnamese coast and from the French coast were maintained at 23°C and 16°C, respectively. The cultures were illuminated using cool-white fluorescent tubes (at a photon flux density of 300 *μ*mol photons PAR m^−2^ s^−1^, Philips TLD, 18 W) under a light-dark (14/10 h) cycles. The photon flux density was measured using a 4*π* waterproof light probe (Walz, Germany) connected to a Li-Cor 189 quantum meter. The growth temperatures were maintained for measurements. For experiments, exponentially growing cells were harvested from precultures, centrifuged gently (900 ×g, 10 min, 4°C) and inoculated sterilely into fresh ASW supplemented or not with a sterile ZnCl_2_ stock solution. The final Zn concentration was 20 *μ*M. The Zn concentration of fresh ASW was 0.25 *μ*M. The cultures were performed in Erlenmeyer flasks of 250 mL capacity that were inoculated at a cell density of 10^4^ cells  mL^−1^. This concentration was chosen after preliminary trials showing that this Zn concentration was the highest Zn concentration tolerated by all four diatoms for at least 10 days (results not shown). All the measurements were performed with cells from cultures at the exponential growth phase that is 5 days from inoculation (data not shown).

### 2.2. Algal Growth and Chlorophyll a and c Contents

Growth in the cultures was monitored by daily cell counts using a Neubauer type hemacytometer. The growth rate was calculated during the exponential phase, and the maximum cell density was determined from the stationary phase of the growth curves. Chlorophyll (Chl) *a *and Chl *c* were measured spectrophotometrically according to Speziale et al. [22].

### 2.3. Oxygen Evolution and Chlorophyll Fluorescence Measurements

Oxygen evolution was determined using a thermostated chamber equipped with a Clark-type oxygen electrode (DW2, Hansatech Instruments Ltd., UK). The oxygen evolution was measured under actinic irradiance ranging from 0 to 1200 *μ*mol photons PAR m^−2^ s^−1^. The gross photosynthesis was calculated as the net photosynthesis plus respiration, assuming that the respiration rate was constant in light and in darkness. The gross photosynthesis versus irradiance curves (*P* versus *E* curves) were fitted according to the model of Eilers and Peeters [23] using the Sigma-plot software.

Chl fluorescence was measured using a FMS1 modulated fluorometer (Hansatech Ltd., UK) modified to make it suitable for use at low Chl *a* concentrations [24]. To obtain the relative electron transport rate versus irradiance (rETR versus *E*) curves, algae were submitted to 11 levels of actinic light progressing from 0 to 1200 *μ*mol photons PAR m^−2^ s^−1^. The fitting of experimental data to rETR versus *E* curves were calculated as indicated by Eilers and Peeters [23] and Mouget et al. [25].

### 2.4. Carbonic Anhydrase Activity

The carbonic anhydrase (CA) activity was measured according to Dionisio-Sese and Miyachi [26] and Morant-Manceau et al. [27]. Intact cells were used to quantify the extracellular CA activity (CA_ext_), while the total CA activity was quantified using cells homogenized in liquid nitrogen (CA_tot_). The internal CA activity (CA_int⁡_) was calculated as CA_tot_ activity minus CA_ext_ activity.

### 2.5. Antioxidant Enzymatic Activities

The algae were harvested by centrifugation (900 ×g, 4°C) and ground in a liquid nitrogen frozen potassium phosphate buffer (K_2_HPO_4_ 50 mM, EDTA Na_2_ 1 mM, pH 7) using a mortar and a pestle. The homogenate was centrifuged (10,000 ×g, 15 min, 4°C), and the supernatant was used for spectrophotometric determination of enzymatic activity. Catalase (CAT) activity was estimated by tracking the reduction of H_2_O_2_ at 240 nm and 20°C [28]. The reaction mixture contained 200 *μ*M H_2_O_2_ in 50 mM of pH 7.5 potassium phosphate buffer. Ascorbate peroxidase (APX) activity was evaluated by tracking the changes in absorbance at 290 nm of the ascorbate substrate in a reaction mixture composed of ascorbate 10 mM and H_2_O_2_ 10 mM in 50 mM of pH 7.0 potassium phosphate buffer. Ascorbate oxidation was measured at 25°C [29]. One unit of enzymatic activity (CAT and APX) was defined as the amount of enzymes that catalyses the conversion of one *μ*mole of substrate per min [30]. Superoxide dismutase (SOD) activity was determined by measuring the inhibition of photochemical reduction of nitroblue tetrazolium (NBT), which absorbs at 560 nm. One unit of SOD activity was calculated as the amount required to cause 50% inhibition of the photoreduction of NBT [31]. Protein concentration of diatom extracts was determined by standardizing versus bovine serum albumin, according to Hartree [32].

### 2.6. Extraction of Nucleic Acids, PCR Amplification, and Bacterial Transformation

DNA was extracted from about 1 g of fresh tissues as described by J. J. Doyle and J. L. Doyle [33] after grinding in liquid nitrogen. The samples were dissolved in 80 *μ*L water. Partial genomic DNA sequences of phytochelatin synthase were obtained by the following PCR procedure. Primer sequences FPCdia/RPCdia (5′-ATGGAARGGACCATGGAGRTG-3′ and 5′-ATRGGWGAAAAATGYCCMGTTCC-3′) and nested primer sequences NFPCdia/NRPCdia (5′-ACCATGGAGRTGGTAYGARGA-3′ and 5′-TTCCAGTTTGMCC-3′) corresponding to conserved sequences were designated from the alignment of PCS nucleic acid sequences of both model diatoms: *Thalassiosira pseudonana* and *P. tricornutum* (http://genome.jgi-psf.org/). Thirty cycles consisting of denaturing for 30 s at 94°C, annealing for 1 min at 57.2°C, and extension for 2 min at 72°C were performed. The reaction was completed by an extension step at 72°C. The first PCR was performed with 0.2 *μ*M of FPCdia, 0.2 *μ*M of RPCdia, and 2.5 units of *Thermus aquaticus* (Taq) DNA polymerase (Promega). Amplified DNA products were subjected to a second PCR with nested primers using the same conditions, apart from a slightly higher annealing temperature (57.5°C). PCR products were cloned into pGEMT-Easy vector (Promega) containing a cassette conferring the resistance to ampicillin. The ligation productions were transformed into *Escherichia coli* DH5*α*. Recombinant bacteria were selected and sequenced on both strands (Operon, Deutschland). Total RNAs (control and sample with Zn 20 *μ*M) were extracted using the RNeasy Plant Mini Kit (Qiagen, MD, USA), and stored at −80°C before northern blot analysis.

### 2.7. Sequence Analysis

The sequences obtained after PCR were subjected to a homology search through the BLAST program available at the NCBI GenBank biocomputing site (http://blast.ncbi.nlm.nih.gov/Blast.cgi) [34]. The deduced amino acid sequences were obtained using the translate software available at the server: (http://www.bioinformatics.org/sms/index.html). The multiple alignments of the sequenced fragments were carried out using the ClustalW EBI program and visualized using Genedoc, version 2.6 [35].

### 2.8. Northern Blot Analysis

Equal amounts (7.5 *μ*g) of total RNA samples were denatured and fractionated by electrophoresis in 1.2% agarose denaturing gel [36]. Total RNA quality was confirmed by ribosomal RNA integrity observed after agarose gel ethidium bromide treatment [36]. Gels were blotted by a capillary procedure [36] on NY Plus membrane (Porablot, Macherey-Nagel, Düren, Germany). Fractionated RNAs were crosslinked at 80°C. The membrane was stained with methylene blue to check the ribosomal RNA quality. The radiolabeled *PCS *probe was obtained by using the Prime a Gene Labeling System kit (Promega, Madisson, WI, USA) with the cloned cDNA adding 50 *μ*Ci (330 nM) of [*α* ^32^P]dCTP. The probe was purified on G50 microcolumns (Amersham-Pharmacia, Orsay, France). Membranes were prehybridized in a hybridization buffer [36] for 2 h, and then [*α* ^32^P]dCTP radiolabeled probes 1 × 1010 cpm *μ*g^−1^  were added. Membranes were exposed to X-ray film (Kodak) for 12 h at  –70°C. These experiments were duplicated.

### 2.9. Statistical Analysis

We used a one-way analysis of variance (ANOVA) to determine the statistical significance of differences in all experiments. To be statistically significant, a difference had to display a level of significance of at least 5% (*P* ≤ 0.05) using the Tukey test run on *SigmaStat version 3.1 *software compatible with *SigmaPlot 9.0*. All measurements were made on 3–5 replicates (from different cultures), and the results were expressed as means and standard errors.

## 3. Results and Discussion

### 3.1. Effects of Zinc on Growth

In the absence of Zn-supplementation, the highest cell density was reached with *N. palea, *which was also the taxon dividing with the slowest rate (Table 1). The two *Amphora *taxons behaved similarly, reaching medium cell densities but the highest dividing rate. *E. paludosa *reached the lowest cell density and the division rate was intermediate between those measured for *Amphora sp. *and *N. palea *(Table 1). These data agree with those published previously on the same *Amphora *species but not for *N. palea *and *E. paludosa, *for which higher values were found by Nguyen-Deroche et al. [10]. The supplementation of the growth medium with Zn affected differentially the growth of the different taxons. For the four taxons, the maximum cell density decreased, while the growth rate remained constant in the *Amphora* species, increased in *N. palea, *and dramatically decreased in *E. paludosa *(Table 1). Altogether, the data suggests that in *N. palea, *Zn stimulated mitosis for a short period before to inhibit this process, leading to a reduced maximum cell density. In the other taxons, Zn ions have only negative effects on culture growth. This negative effect has been already observed for lower Zn concentrations in different species such as *Nitzschia closterium* (0–1.52 *μ*M: [6]), *S. costatum *(24 pM: [37]), and *P. tricornutum* (0.05–10 *μ*M: [38]).

The results of this experiment allowed us to range both *Amphora *species as Zn-tolerant taxons and both *P. paludosa *and *N. palea *as Zn-sensitive taxons. This conclusion fits with the results already published on Zn sensitivity of *Nitzschia* [6]. Interestingly, these taxons reacted differently when facing to an increase of Cu [10]. Despite the fact that Zn can be important for mitosis regulation [4], algal growth depends primarily on photosynthesis. Therefore, this process was characterized at the biochemical and physiological level in the four diatom species grown in the presence or in the absence of Zn.

### 3.2. Effects of Zinc on Chlorophyll Contents

Chl quantifications in the four taxons grown in the absence of the Zn-supplementation revealed that *A. acutiuscula *and *E. paludosa *contained three times more total Chl than the two other taxons. Chl *a* was always the major pigment (Table 1). This difference was not reflected in the Chl *a*/Chl *c* ratio, always higher than 8, except for *N. palea *for which the ratio was close to 5. Because the Chl *a*/Chl *c* ratio constitutes a rough measure of the size of the light-harvesting antenna [39], this result suggests that the antenna of *N. palea* is larger than in the other species. The addition of Zn did not significantly impact the total Chl amount in *A. acutiuscula, *whereas it triggered an increase in *N. palea* and a decrease in *A. coffeaeformis *and *E. paludosa*. The Chl *a*/Chl *c* ratio was not affected in *A. acutiuscula *and* N. palea, *whereas it was decreased by at least two units in *E. paludosa* and *A. coffeaeformis *(Table 1). Although the different culture protocols used in the literature make difficult the comparison of Zn effects on Chl contents, it is generally found that metals in excess, including Zn, reduce the Chl *a* amount (Zn-*Chlorella vulgaris*: [40]; Zn-*Pavlova viridis*: [11]; Cd, Cu-multispecies: [41]) with a notable exception in the diatom *Asterionella japonica* for which an increase was reported [42]. The way used by Zn to impact the Chl amount is not clear and no reasonable hypothesis can be proposed at the present state of our knowledge. Regardless of this reason, it is worse to mention that the Chl *a*/Chl *c* ratio remained stable while in green algae, the ratio Chl *a*/Chl *b* decreases due to the inhibition of Chl *b *formation from Chl *a* [43, 44]. Because Chl *c* derived from the Chl precursor protochlorophyllide (reviewed in [45]), any block or stimulation of the biochemical steps prior protochlorophyllide formation would affect similarly the amount of both pigment types leading the ratio to remain unchanged. Altogether, the results suggest that the Zn excess does not modify the size of the light-harvesting complexes except in *E. paludosa *and in *A. acutiuscula*. In the two other species, the increase in total Chl content would speak in favor of a Zn-induced increase of the number of photosynthetic chains. In order to test this hypothesis, we measured the variation of the gross photosynthesis and of the relative electron transfer rate versus the irradiance level.

### 3.3. Effects of Zinc on Photosynthesis

**Figure 1:**
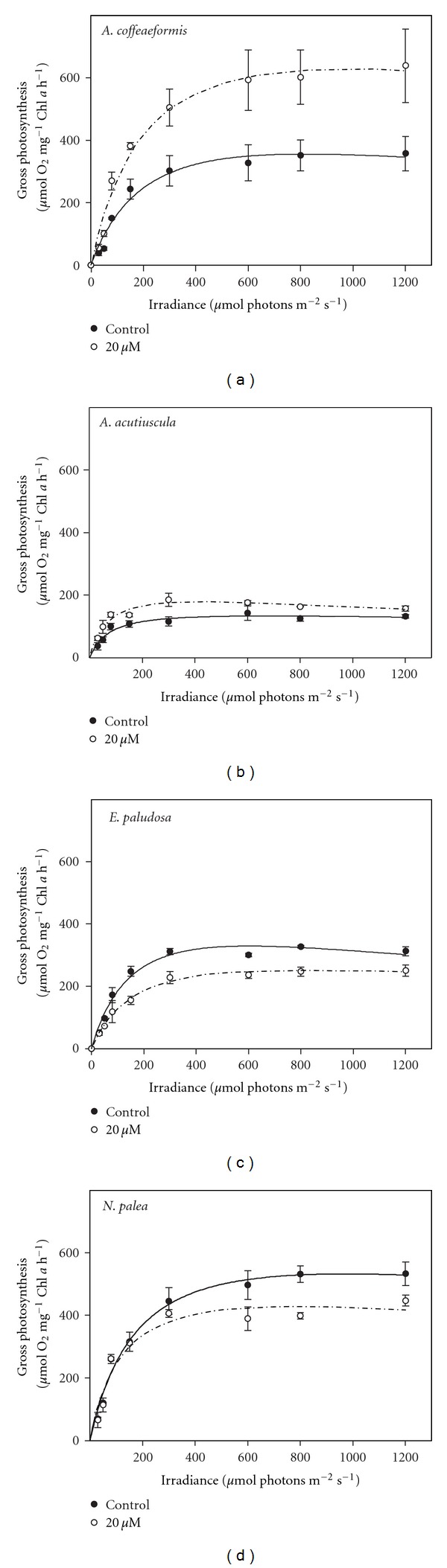
Gross photosynthesis versus irradiance curves in *Amphora coffeaeformis*,* Amphora acutiuscula, Entomoneis paludosa,* and *Nitzschia palea* grown in ASW (control) or in the presence of 20 *μ*M Zn added to ASW. Mean values ± SE (*n* = 3–5).

In the absence of Zn-supplementation, the curves *P/E* presented the same trends. Both increased and saturated between 600–800 *μ*mol photons PAR m^−2^ s^−1^ (Figure 1). However, the maximum amplitude reached was different for the different species (Table 2). The Zn-supplementation affected negatively the gross photosynthesis in *E. paludosa *and* N. palea *but positively that of both *Amphora* species (Figure 1), confirming that these species are better in managing the excess of zinc. A decrease in photosynthetic activity has also been observed in other microalgae at various Zn concentrations (*S. costatum*- >24 pM: [37]; *Chlamydomonas reinhardtii*-30.8 *μ*M: [46]; *Pseudokirchneriella subcapitata*-14 *μ*M: [47]).

The impairment of photosynthesis is reflected in the values of the parameters characterizing *P/E* curves (Table 2).

**The *α* ^*B*^ Parameter**

**Figure 2:**
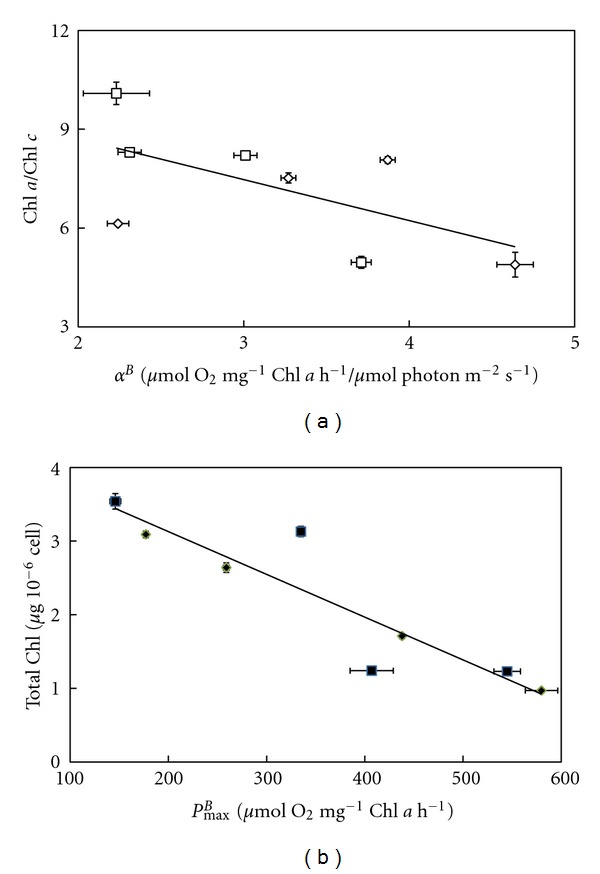
(a) Relationship between *α* ^*B*^ calculated from gross photosynthesis versus light intensity curves (*P/E* in Figure 1) and Chl *a*/Chl *c* ratio (Table 1) in *Amphora coffeaeformis, Amphora acutiuscula, Entomoneis paludosa,* and *Nitzschia palea* grown in ASW in the absence (□) or the presence (*◊*) of Zn supplementation. (b) Relationship between the total Chl content (Table 1) and the maximum gross photosynthesis (*P*_max⁡_^*B*^) (Figure 1) in *Amphora coffeaeformis, Amphora acutiuscula, Entomoneis paludosa,* and *Nitzschia palea* grown in ASW in the absence (■) or the presence (*◆*) of Zn supplementation.

It reflects the affinity of the algae for light. In the absence of Zn-supplementation, *α* ^*B*^ ranged between 2.2-2.3 for the *Amphora *species to 3.0–3.7 for the two other species. These values were higher in the presence of the Zn-supplementation except in *E. paludosa* for which it decreased. For the same photon flux density, the speed at which O_2_ is evolved is primarily dependent on the size of the light harvesting complex, which is reflected in the Chl *a*/Chl *c* ratio. Therefore, a linear relationship between the two parameters should be observed. To test this hypothesis, the *α* ^*B*^ values were plotted against the Chla/Chlc values obtained with diatoms grown in the presence of an excess of Zn (Figure 2(a)). The linear relationship obtained suggests the validity of the hypothesis.

**The *P*_max⁡_^*B*^ Parameter**

This factor reflects the photosynthetic activity when the light is saturating. In the absence of Zn-supplementation, the values of *P*_max⁡_^*B*^ were high except for *A. acutiuscula* for which the value was reduced by 50 to 75% (Table 2). *P*_max⁡_^*B*^ was increased in both *Amphora* species, but was lower in the other two diatoms in comparison to controls. The maximum oxygen evolved is primarily related to the total Chl present and therefore a linear relationship should be found when the *P*_max⁡_^*B*^ values are plotted against the total Chl amount. This linear relationship was indeed found (Figure 2(b)).

**The Parameter *E*_*k*_**

It reflects the photon flux density from which the photosynthetic activity does not increase proportionally to the light intensity. In the absence of  Zn supplementation, the values of *E*_*k*_ for *A. acutiuscula* and *E. paludosa *were lower than those obtained for the two other species suggesting that the two former species are more sensitive to high-light than the others. This is also reflected by the lower value of *P*_max⁡_^*B*^ for these two species. The Zn supplementation did not change significantly the *E*_*k*_ levels (Table 2).

**Figure 3:**
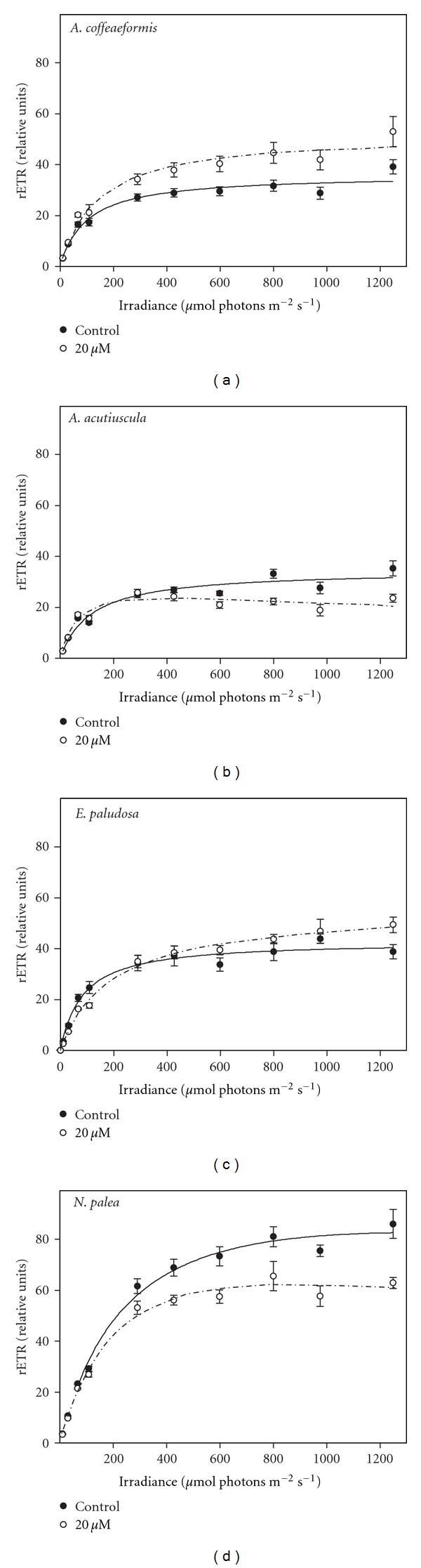
Relative electron transport rate (rETR) versus irradiance curves in *Amphora coffeaeformis*, *Amphora acutiuscula, Entomoneis paludosa,* and *Nitzschia palea* grown in ASW (control) or in the presence of 20 *μ*M Zn added to ASW. Mean values ± SE (*n* = 3–5).

The *P/E* curves give information on how Zn affects the PSII functioning. In order to enlarge our picture on the impact of Zn on the photosynthetic process, we followed the response of the relative electron transport rate (rETR) to increasing photon flux density. In the absence of Zn-supplementation, the curves rETR/*E* presented the same trends as the *P/E* curves except that they never completely saturated. rETR_max⁡_ reached were similar among the different species (around 40) except for *N. palea*, which reached 80 (Figure 3). The Zn-supplementation affected negatively the rETR in *E. paludosa *and* N. palea*, suggesting that Zn might have several targets. To get more information from the curves, the characteristic parameters were calculated (Table 2).

**The *α*_rETR_ Parameter**

It reflects the efficiency of the algae to use the incoming light to drive the electron transport. In the absence of Zn-supplementation, the taxons were equally performant in using the incoming light except *A. acutiuscula*, which was the less efficient. In *N. palea *and *A. coffeaeformis*, *α*_rETR_ was not modified, whereas it was considerably higher in *A. acutiuscula *(+95%) and lower in *E. paludosa* (−40%).

**The rETR_max⁡_ Parameter**

This factor reflects the maximum ETR when the light is saturating. In the absence of Zn-supplementation, the taxons reached the same value for this parameter except *N. palea*, which exhibited a much higher level at saturation. In the presence of Zn excess, the intensity of this parameter significantly increased in *A. coffeaeformis *and *E. paludosa, *whereas it significantly decreased in the two other species.

**The *E*_*k*rETR_ parameter**

It reflects the photon flux density from which the ETR does not increase proportionally to the light intensity. *E. paludosa *and *A. coffeaeformis *presented lower values than for the two other species. The values of this parameter were reduced in *N. palea *and *A. acutiuscula*, but considerably increased in *E. paludosa *(+107%) (Table 2).

**Figure 4:**
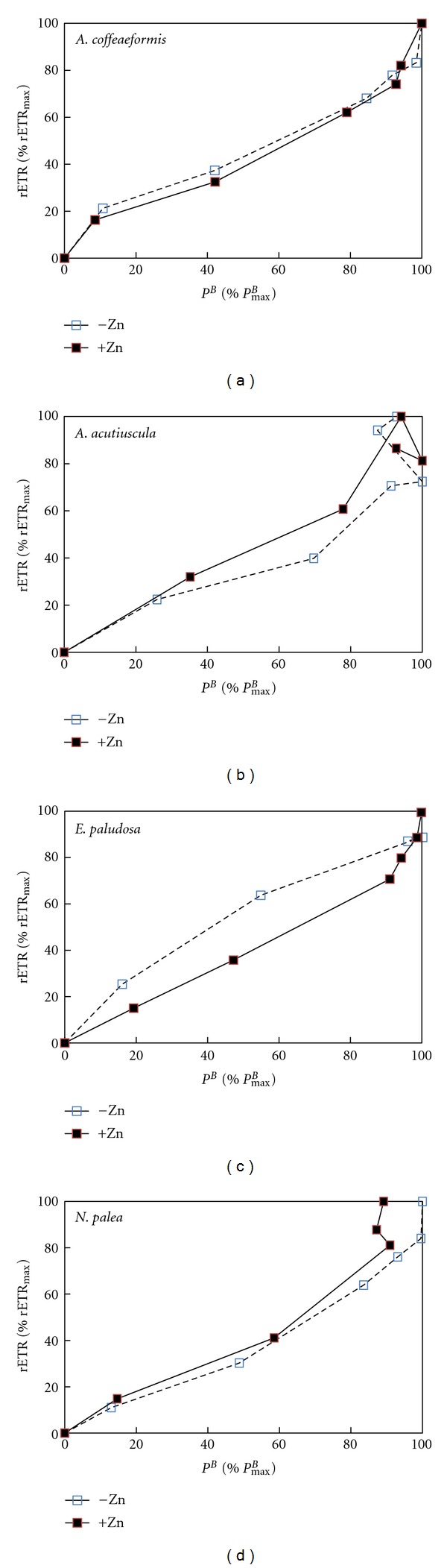
Relationship between the relative intensity of rETR and the relative intensity of *P* ^*B*^ in* Amphora coffeaeformis, Amphora acutiuscula, Entomoneis paludosa,* and *Nitzschia palea* grown in the absence (□) or the presence (■) of a Zn supplementation.

*P/E* and rETR/*E* are two ways to measure the photosynthetic activity [48]. Therefore, from the theoretical point of view, both parameters vary in the same way [49] as shown in the case of *A. coffeaeformis* in the absence or in the presence of Zn supplementation (Figure 4). However, a stress may affect differentially the PSII and the electron transport chain and disrupts the linear relationship between these two parameters. This is obviously the case in *A. acutiuscula *(Figure 4), in which the absence of Zn made the electron rate slower than the oxygen evolution rate. The Zn supplementation restored the proportionality between the two activities. This result suggests that in the ASW used here, *A. acutiuscula *underwent a slight Zn deprivation. This slight Zn deprivation would also affect *A. coffeaeformis* because both parameters were most intense in the presence of Zn (Figures 1 and 3).

In the absence of Zn, the electron transport rate was faster than the oxygen evolution rate in *E. paludosa*. Such a behavior could be explained by the involvement of other mechanisms such as Mehler reaction, cyclic electron transport around PSII and/or PSI, photorespiration, and light-dependent mitochondrial respiration. The intensity of these mechanisms depends on the experimental conditions [50]. We observed that the Zn supplementation restored the proportionality between the two parameters, suggesting that Zn may target some component(s) of the electron transfer chain (Figure 4). The cytochrome of the electron transport chain can be proposed as a putative target of Zn ions in excess. Actually, it has been shown that Zn ions interact with the Q_0_ pocket of cytochrome b_6_/f complex [51]. These ions have also been shown to impair the proton transport function of cytochromes in bacteria and mitochondria [52]. Because the structure of cytochrome has been highly conserved during evolution [53, 54], this possibility is also likely.

In *N. palea, *Zn slowed down both oxygen evolution and the electron transport rates, with the rETR being more impacted at the highest photon flux densities (>800 *μ*mol photon PAR m^−2  ^ s^−1^) than the oxygen evolution rate. Several nonexclusive causes can be involved in this inhibition: (i) *photoinhibition due to a reduced activity of the xanthophyll cycle*: the cycle consists in the reversible conversion of diadinoxanthin to diatoxanthin. It is activated by the acidification of the thylakoid. It is used as a photoprotection mechanism allowing the dissipation of the excess of energy absorbed by PSII. When this capacity is over, the photoinhibition starts [55, 56]. In our conditions, an impairment of the xanthophyll cycle is unlikely as no photoinhibition was observed (Figures 1 and 3). If this phenomenon would occur, both *P/E* and rETR/*E* curves would have presented a strong decreasing phase at high photon flux densities. So far the only metal known to inhibit the xanthophyll cycle activity in diatoms is cadmium [57]. (ii) *PSII inhibition*: it can be due to the impairment of the water oxidizing enzymes itself or/and by the destabilization of the binding cofactors in the oxygen evolving polypeptides associated with PSII [58]. For instance, Vaillant et al. [59] established that the replacement of Mn^2+^ in the water oxidizing complex by Zn^2+^ leads to a reduction of oxygen emission. Altogether these data indicate that in *N. palea*, the reduction of photosynthetic activity triggered by the excess of Zn explains the lower maximum cell density presented in Table 1, with the cell becoming at this Zn concentration unable to cope with its toxicity. (iii) *A shortage of carbon supply: *Subrahmanyam and Rathore [60] found that a reduced demand for ATP and NADPH in the Calvin cycle causes a downregulation of PSII photochemistry. On the other hand, Sunda and Huntsman [61] have identified a relationship between the addition of Zn and the C fixating rate at saturating light intensity in the diatom *Thalassiosira pseudonana* and in higher plants, Zn can inhibit the carboxylase activity of RuBisCO, leading intact the oxygenase capacity [62].

In diatoms, carbonic anhydrase, a Zn-dependent enzyme catalyses the reversible interconversion of HCO_3_^−^ and CO_2_ and is an important component of the inorganic carbon concentration mechanism [63–65]. This enzyme supplies RubisCO with CO_2_ [27, 66]. The positive effects of Zn on the photosynthetic activity of *A. coffeaeformis *suggest that the amount of Zn in the ASW constitutes a limiting factor (Figures 1 and 3) that could limit the CA activity. In order to test this hypothesis, the effect of Zn-supplementation on the CA activity was measured. These data are presented in the next section.

### 3.4. Effects of Zinc on Carbonic Anhydrase Activity

**Figure 5:**
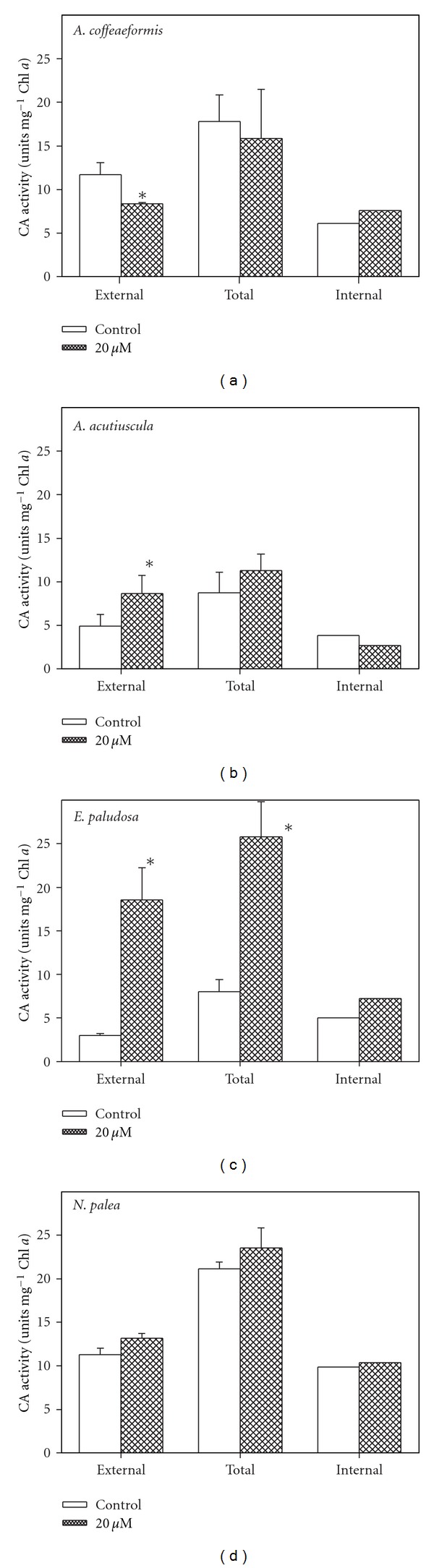
External, internal, and total carbonic anhydrase (CA) activities in* Amphora coffeaeformis, Amphora acutiuscula, Entomoneis paludosa,* and *Nitzschia palea* grown in ASW (control) or in the presence of 20 *μ*M Zn added to ASW. Mean values ± SE (*n* = 3–5). Significant differences are indicated by an asterisk (*P* ≤ 0.05).

In the absence of the Zn-supplementation, the carbonic anhydrase activity was detected at the cell surface (external CA) and in the cytosol (internal CA) in all four diatoms, with the highest total activity being found in *A. coffeaeformis *and *N. palea*. The addition of Zn did not stimulate CA activity except the CA_ext_ activity in *E. paludosa* (Figure 5). It can be noticed that the weak increase of CA_ext_ activity in *A. acutiuscula *could be reflected in the higher photosynthetic activity (Figures 1 and 3).

It is well established that metal stresses induce the production of ROS that disturbs the functioning of the different cell compartments [15]. To test this possibility in our growth conditions, the total activity of the main antioxidant enzymes that is, SOD, APX, and CAT were measured after 5 days of growth in the presence or the absence of Zn-supplementation.

### 3.5. Antioxidant Enzymatic Activities

**Figure 6:**
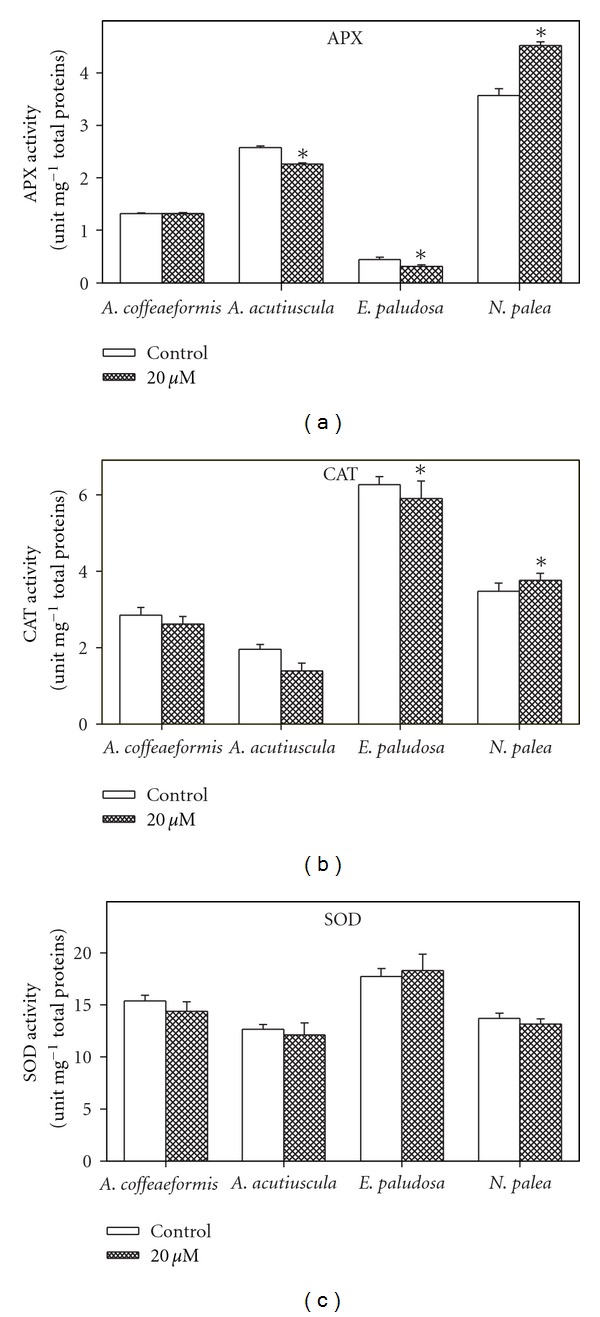
Antioxidant enzymes activities (superoxide dismutase, SOD; catalase, CAT and ascorbate peroxidase, APX) in* Amphora coffeaeformis, Amphora acutiuscula, Entomoneis paludosa,* and *Nitzschia palea* grown in ASW (control) or in the presence of 20 *μ*M Zn added to ASW. Significant differences are indicated by an asterisk (*P* ≤ 0.05). Mean values ± SE (*n* = 3–5).

Each taxon presented an activity APX, CAT, and SOD in the absence of Zn-supplementation but with different relative intensities (Figure 6). In the four species, the SOD activity represented about 70% the total antioxidant activity measured, the remaining activities being shared unequally between APX and CAT activities. For instance, in *E. paludosa, *the CAT activity was 12 times higher than the APX one (Figure 6).

 In the Zn-supplemented growth medium, the activity of the three antioxidant enzymes did not display any clear increase, except the APX activity in *N. palea* that increased by 22%. However, we could not exclude the possibility that the activity of the enzymes is modified in individual cell compartments, such as the chloroplasts, in which the ROS production can elevate in case of photosynthetic impairment (reviewed in [5]), these results presented here suggest that in our conditions, the excess of Zn did not triggered an intensive oxidative stress requiring additional antioxidative enzymes to cope with Pinto et al. [67] have shown that in *Pavlova viridis* an excess of Zn (*c.a.* 50 *μ*M) enhanced lipid peroxidation, which can be considered as an indication of the oxidation damages. Alternatively, we can suggest that a part of the ions in excess is quenched, with the remaining part being unable to trigger an intense oxidative stress. So far, two main mechanisms of ion quenching have been found to be active in algae, including diatoms (reviewed in [5, 15]). The first mechanism occurs outside the cells and involved the binding of the metal ions to exopolysaccharides (Zn-*S. costatum*: [16]; Cu-*Amphora *sp.: [10]). Although these exopolysaccharides were not quantified in this study, we observed that the four diatoms tended to agglutinate when placed in the Zn-supplemented medium (data not shown), suggesting the production of these compounds as reported in *A. coffeaeformis *[68]. However, the binding capacity of the exopolysaccharides seems not intense enough to avoid Zn penetrating into the cells. The second mechanism occurs mostly in the cytoplasm and consists in the phytochelatins (Cu, Zn*-Scenedesmus *sp.: [31]; Zn-*Nitzschia closterium*: [6]) (reviewed in [67]). In order to test the second possibility, the genes corresponding to phytochelatin synthase were searched and their expression was measured in the different taxons grown in the presence or in the absence of Zn-supplementation.

### 3.6. Partial Phytochelatin Synthase Sequences

**Figure 7:**
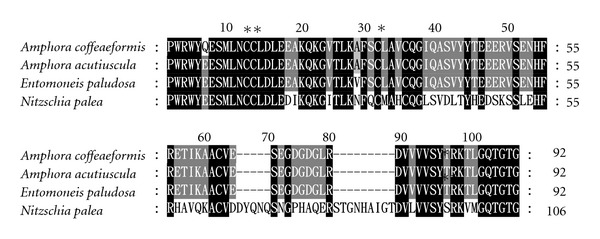
Alignment of the amino acid sequences of phytochelatin synthase fragments isolated from *Amphora acutiuscula *(FN995989),* Amphora coffeaeformis *(FN995985),* Entomoneis paludosa *(FN995987) and *Nitzschia palea *(FN995985) grown in ASW. Black boxes indicated 100% identity, dark grey 80%, and light grey 60%. The cysteine residues are indicated by asterisks.

The use of the designed primers allowed the recovery of the partial DNA sequences in each taxon studied. The DNA sequence analysis showed open reading frames ranging from 279 to 321 bp (data not shown) coding for 92 to 106 amino acids residues, respectively, for the four taxons investigated in this study (Figure 7). Blast searches using the nucleotide sequences against those of higher plants as well as the sequenced genomes of the diatoms *P. tricornutum *and *T. pseudonana *revealed identities up to 100% with phytochelatin synthase gene (98%: *E. paludosa* and both *A. coffeaeformis*; 100%: *A. acutiuscula*). The *in silico *translation of the open-reading frames revealed the presence of four conserved cysteine residues belonging to the catalytic domain located at the N-terminal region of the enzyme [69, 70] (Figure 7). Both these DNA sequences and the corresponding deduced amino acid sequences have been deposited to the EML-EBI database (*N. palea* no. FN995985; *A. coffeaeformis*, no. FN995986; *E. paludosa*, no. FN995987; *A. acutiuscula*, no. FN995989). To evaluate the expression level of the phytochelatin synthase gene in the absence and in the presence of Zn-supplementation, total RNA were extracted after 5 days of growth in the absence or the presence of Zn-supplementation and quantified by northern blotting. The good quality of the total RNA extracted was revealed by two clearly defined electrophoretic bands corresponding to 18S and 28S ribosomal RNA (data not shown). Despite the fact that Zn is the second best inducer of phytochelatin synthesis [71], the mRNAs corresponding to phytochelatin synthase were only detected in equal amount in *N. palea* in the presence or in the absence of Zn-supplementation (data not shown), and using this method, no change in the expression level was suspected due to the presence of the Zn supplementation. Interestingly, the diatom *P. tricornutum* did not express phytochelatin synthase for a Zn concentration one order lower than our (2.2 *μ*M: [72]). On the other hand, Zn has been reported to trigger phytochelatin synthesis in the green alga *Dunaliella tertiolecta* for a Zn concentration one order higher than the one used in this study (200 *μ*M: [73]). This suggests that each taxon would sense the Zn internal concentration and would express the phytochelatin synthase gene according to a threshold, with this level being one ecological characteristic of this taxon. Our data suggest that this minimum level was crossed only in the case of *N. palea. *The synthesis of phytochelatins would then contribute to the resistance of *N. palea* in Zn-supplemented growth medium. This result also confirms that this taxon is especially sensitive to Zn elevation. More investigations are needed to find out whether the phytochelatin synthase genes are completely repressed or slightly expressed in the other three species.

## 4. Conclusions

A Zn supplementation to the growth medium has different effects on the metabolism of diatoms. Of the four diatoms tested, *E. paludosa* was found to be the most sensitive taxon to Zn supplementation since its growth is drastically decreased. This study also showed that Zn in ASW is a limiting factor for both *Amphora *species. *A. coffeaeformis *is the most tolerant species in our culture condition. In *N. palea, *a higher antioxidant enzyme activity and the expression of phytochelatin gene are mechanisms providing cellular tools to cope with the excess of Zn and allowing the cells to develop equally to the tolerant species *A. coffeaeformis*.

## Figures and Tables

**Table 1:** Growth rate, maximum cell density, chlorophyll *a* and *c* contents, total Chl, Chl *a*/Chl *c* ratio *in Amphora acutiuscula, Amphora coffeaeformis, Nitzschia palea, *and* Entomoneis paludosa* grown in ASW (control) or in the presence of Zn 20 *μ*M added to ASW.

Species	*A. acutiuscula*		*A. coffeaeformis*		*N. palea*		*E. paludosa*	
	Control	Zn 20 *μ*M	Control	Zn 20 *μ*M	Control	Zn 20 *μ*M	Control	Zn 20 *μ*M
Growth rate (day^−1^)	1.210 ± 0.074^a^	1.281 ± 0.014^a^	1.198 ± 0.192^a^	0.957 ± 0.108^a^	0.258 ± 0.028^a^	0.405 ± 0.069^b^	0.630 ± 0.430^a^	0.020 ± 0.017^b^
Maximum cell density (10^3^ cells mL^−1^)	591± 8^a^	498 ± 5^b^	617 ± 4^a^	480 ± 4^b^	1308 ± 133^a^	627 ± 8^b^	298 ± 14^a^	48 ± 1^b^
Chl* a *(*μ*g 10^−6^ cells)	3.22 ± 0.20^a^	2.72 ± 0.20^b^	1.10 ± 0.03^a^	0.87 ± 0.03^b^	1.03 ± 0.05^a^	1.42 ± 0.01^b^	2.79 ± 0.36^a^	2.27 ± 0.10^a^
Chl* c *(*μ*g 10^−6^ cells)	0.32 ± 0.01^a^	0.37 ± 0.03^b^	0.13 ± 0.01^a^	0.11 ± 0.01^a^	0.21 ± 0.02^a^	0.29 ± 0.01^b^	0.34 ± 0.06^a^	0.37 ± 0.03^a^
Total Chl (*μ*g 10^−6^ cells)	3.54 ± 0.21^a^	3.09 ± 0.09^a^	1.24 ± 0.04^a^	0.97 ± 0.03^b^	1.23 ± 0.07^a^	1.71 ± 0.22^a^	3.13 ± 0.42^a^	2.64 ± 0.13^a^
Chl *a*/Chl *c *	10.09 ± 0.40^a^	7.52 ± 0.75^b^	8.30 ± 0.14^a^	8.07 ± 0.09^a^	4.96 ± 0.12^a^	4.89 ± 0.22^a^	8.2 ± 0.40^a^	6.13 ± 0.15^a^

Mean  values ± SE (*n* = 3–5). Significant different data are indicated with different superscripted letters (Tukey Test, *P* ≤ 0.05).

**Table 2:** Parameters (*α* ^*B*^, light utilization coefficient; *P*_*max*_^*B*^, maximum gross photosynthesis; *E*_*k*_, irradiance for the light saturation of photosynthesis) of gross photosynthesis versus irradiance curves. *α* ^*B*^: **μ**mol *O*_2_ mg^−1^ Chl *a* *h* ^−1^ (*μ*mol photons m^−2^ s^−1^)^−1^; *P*_*max*_^*B*^: *μ*mol O_2_ mg^−1^ Chl *a* *h* ^−1^; *E*_*k*_: *μ*mol photons m^−2^ s^−1^. Parameters (*α*_*rETR*_, light utilization coefficient; *rETR*_*max*_, maximum relative electron transport rate; *E*_*krETR*_, irradiance for the light saturation of photosynthesis) of relative electron transport rate versus irradiance curves. *α*_*rETR*_/rETR (*μ*mol photons m^−2^ s^−1^)^−1^; *rETR*_*max*_: relative units; *E*_*krETR*_: *μ*mol photons m^−2^ s^−1^.

Species	*A. acutiuscula*		*A. coffeaeformis*		*N. palea*		*E. paludosa*	
	Control	Zn 20 *μ*M	Control	Zn 20 *μ*M	Control	Zn 20 *μ*M	Control	Zn 20 *μ*M
*α* ^B^	2.23 ± 0.68^a^	3.27 ± 0.31^b^	2.31 ± 0.05^a^	3.87 ± 0.20^b^	3.71 ± 0.36^a^	4.64 ± 0.75^a^	3.01 ± 0.14^a^	2.24 ± 0.13^b^
*P*_max⁡_^*B*^	146 ± 11^a^	177 ± 3^b^	407 ± 44^a^	580 ± 33^b^	545 ± 27^a^	438 ± 5^b^	335 ± 2^a^	259 ± 6^b^
*E*_*k*_	88 ± 29^a^	55 ± 4^a^	175 ± 15^a^	178 ± 18^a^	153 ± 21^a^	102 ± 16^a^	110 ± 5^a^	116 ± 4^a^
*α*_rETR_	0.21 ± 0.02^a^	0.41 ± 0.03^b^	0.36 ± 0.03^a^	0.36 ± 0.01^a^	0.42 ± 0.02^a^	0.39 ± 0.03^a^	0.48 ± 0.05^a^	0.29 ± 0.01^b^
rETR_max⁡_	37 ± 1^a^	24 ± 1^b^	36 ± 1^a^	49 ± 2^b^	95 ± 7^a^	62 ± 3^b^	43 ± 3^a^	53 ± 3^b^
*E*_*k*rETR_	187 ± 28^a^	62 ± 7^b^	112 ± 9^a^	139 ± 8^a^	224 ± 7^a^	166 ± 19^b^	90 ± 6^a^	181 ± 8^c^

Mean  values ± SE (*n* = 3–5). Significant different data are indicated with different superscripted letters (Tukey Test, *P* ≤ 0.05).
